# Recent Interventions for Acute Suicidality Delivered in the Emergency Department: A Scoping Review

**DOI:** 10.5811/westjem.18640

**Published:** 2024-10-09

**Authors:** Alex P. Hood, Lauren M. Tibbits, Juan I. Laporta, Jennifer Carrillo, Lacee R. Adams, Stacey Young-McCaughan, Alan L. Peterson, Robert A. De Lorenzo

**Affiliations:** * University of Texas Health Science Center at San Antonio, Department of Emergency Medicine, San Antonio, Texas; † Baylor University, Department of Psychology and Neuroscience, Waco, Texas; ‡ University of Texas Health Science Center at San Antonio, Department of Psychiatry and Behavioral Sciences, San Antonio, Texas; § University of Texas at San Antonio, Department of Psychology, San Antonio, Texas

## Abstract

**Introduction:**

Suicidality is a growing problem in the US, and the emergency department (ED) is often the front line for the management and effective treatment of acutely suicidal patients. There is a dearth of interventions that emergency physicians may use to manage and effectively treat acutely suicidal patients. To the extent that recently described interventions are available for ED personnel, no review has been conducted to identify them. This scoping review is intended to fill this gap by systematically reviewing the literature to identify recently described interventions that can be administered in the ED to reduce symptoms and stabilize patients.

**Methods:**

We conducted a search of PubMed, SCOPUS, and CINAHL in January 2024 to identify papers published between 2013–2023 for original research trialing recent interventions for the effective treatment of suicidality in the ED. We assessed 16 full-text articles for eligibility, and nine met inclusion criteria. Included studies were evaluated for features and characteristics, the fit of the intervention to the ED environment, and interventional efficacy.

**Results:**

Four studies assessed the efficacy of a single dose of the anesthetic/analgesic agent ketamine. Three studies assessed the efficacy of a brief psychosocial intervention delivered in the ED, two of which paired this intervention with the provision of follow-up care (postcard contact and referral assistance/case management, respectively). The remaining two studies trialed a brief, motivational interviewing-based intervention. Included studies had strong experimental designs (randomized controlled trials) but small sample sizes (average 57). Among the interventions represented across these nine studies, a single dose of ketamine and the brief psychosocial intervention Crisis Response Planning (CRP) show promise as ED-appropriate interventions for suicidality. Ketamine and CRP demonstrated the strongest fit to the ED environment and most robust efficacy findings.

**Conclusion:**

This review identified one drug (ketamine) and four unique psychological/behavioral interventions that have been used to treat acute suicidality in the ED. There is currently insufficient evidence to suggest that these interventions will prove efficacious and well-suited to be delivered in the ED environment. Future studies should continue to test these interventions in the ED setting to determine their feasibility and efficacy.

## INTRODUCTION

Over the past two decades, the suicide rate in the US general population increased by over 33%.^1^ Up to half of suicide decedents visit an emergency department (ED) during the year before their death, and approximately 25% visit in the month immediately prior.^2^ ^,^ ^3^ The risk for death by suicide among ED patients presenting with suicidal thoughts and behaviors remains high for at least one year after discharge.^4^ ^,^ ^5^ The ED is often the first medical access point for those with an acute deterioration in their mental health; approximately 10% of all ED visits are for mental health concerns.^5^ ^–^ ^7^ New and innovative approaches are needed to stem the tide of suicides and to help mitigate the crisis of psychiatric boarding in EDs.^8^ ^,^ ^9^

Emergency department personnel have increasingly voiced concerns over a broken system of mental health care that has exacerbated conditions for ED patients with psychiatric emergencies.^10^ Such serious system deficiencies may contribute to the perception of suicidal ED patients who describe ED personnel as lacking empathy, and being brusque, irritable, and even hostile.^11^ Exacerbating the problem is that the number of state-funded inpatient psychiatric beds has dropped substantially, from 340 beds per 100,000 people in 1995 to under 12 beds per 100,000 by 2016.^8^ ^,^ ^9^ Conversely, the number of ED visits for psychiatric complaints has risen by 50%.^8^ This has led to a situation where many patients who require inpatient mental health care must wait in the ED until a psychiatric bed becomes available. This delay in transferring patients to an inpatient unit leads to “psychiatric ED boarding.”^12^

The state-of-the-art interventions available to emergency physicians are oriented toward safely discharging patients home and connecting them to definitive mental health services.^13^ ^,^ ^14^ Brief interventions or referral followed by discharge home are common for patients presenting with non-life-threatening suicidal thoughts and behaviors, whereas patients presenting with moderate to severe risk behaviors for suicide are usually kept in the ED until transfer to an inpatient psychiatric facility is possible.^15^ This splitting of patients into categories of risk severity^16^ means that the higher a patient’s risk for suicide, the fewer interventions are available to address the patient’s particular needs. Notably, no pharmacologic agent has been approved by the US Food and Drug Administration to treat suicidality in the ED; most medications administered to suicidal ED patients typically target only agitation, not the suicidal symptoms themselves.^16^ ^,^ ^17^

From a psychiatric perspective, most available interventions target suicidal thoughts and behaviors over the long term as opposed to the short- or medium term^17^ and are therefore ill-suited to the acute care environment. Psychopharmacologic agents such as antidepressants, lithium, and antipsychotics generally require a course of weeks or months to take effect,^14^ and beginning a course of antidepressant treatment can paradoxically increase suicidality in some populations.^18^ Similar time scales are required for empirically supported psychotherapies such as cognitive behavioral therapy and others,^17^ ^,^ ^19^ and even the most abbreviated standard interventions can take up to six weeks.^20^

While the importance of screening for suicidality is well understood,^21^ there is growing need for evidence-based, rapidly acting, effective treatment options.^15^ Many existing tools suited to the ED environment that target suicidality lack supporting evidence or, worse, are counterproductive.^22^ ^,^ ^23^ One such intervention is the safety contract or no-suicide agreement. While at one time the gold standard for ED anti-suicidal interventions, the safety contract has been shown to produce worse outcomes than no intervention at all.^21^ ^,^ ^24^ To the extent that more recent interventions for the effective treatment of acute suicidality have emerged, there has been no review created specifically to identify and describe potential interventions.

An analysis by Inagaki and colleagues^25^ identified broad classes of interventions to prevent repeat suicide attempts in patients admitted to an ED but did not investigate which interventions would be best suited to the ED environment. Chang and colleagues provided a review of major depressive disorder and suicidality in the ED but did not offer an analysis of recently described interventions.^21^ In a 2021 review, Mann and colleagues^26^ surveyed the landscape for evidence-based therapies for suicidality in general, but they did not focus specifically on the ED. While other recent reviews have assessed the availability of clinician-oriented educational interventions,^23^ or interventions for mental decompensation in general,^27^ none have thoroughly assessed the literature for recently described tools that clinicians may use to treat acute suicidality in the context of the ED. Lengvenyte and colleagues^28^ published a systematic review on the immediate and short-term efficacy of suicide-targeting interventions but did not focus on recent interventions used in the ED. We undertook this review to fill the gap and explore the literature to identify and describe recent, patient-centered interventions for the effective treatment of acute suicidality in the ED.

In this review we focused on recently described interventions that can be administered in the context of a patient’s stay in the ED, namely, brief therapies and pharmacologic agents that fit with the standard medical model of treatment. State-of-the-art practice (ie, generally accepted care), defined as interventions for acute suicidality, are described in *Rosen’s Emergency Medicine: Concepts and Clinical Practice* ^29^ or *Kaplan and Sadock’s* *Comprehensive Textbook of Psychiatry*.^30^ These include screening, joint safety planning, patient education, lethal means counseling, follow-up contacts, and the involvement of friends and family.^29^ ^,^ ^30^ Interventions listed in or moderately modified from those described in these textbooks were considered state-of-the-art and excluded from the search. The primary question of this review was as follows: What recently described interventions are available for use in reducing suicidality and stabilizing patients during a psychiatric crisis in the ED?

## METHODS

We searched PubMed, SCOPUS, and CINAHL on January 15, 2024. This review was conducted in accordance with best-practice recommendations of the Preferred Reporting Items for Systematic Reviews and Meta-Analysis (PRISMA) extension for scoping reviews.^31^ Inclusion criteria included the following: 1) study participant patients were presenting to the ED with suicidal ideation; 2) the study assessed the efficacy of one or more patient-centered intervention(s) aimed at reducing suicidal thoughts and behaviors; 3) the intervention being tested was administered to patients in the ED; 4) the intervention was administered by emergency physicians or personnel; 5) the study was available in English; and 6) the study had been published in the last 10 years.

### Definition of Suicidality and Recent Interventions

We adopted the suicidality nomenclature proposed by Silverman and colleagues.^32^ Studies implementing the broad term suicide-related thoughts and behaviors (SRTB), or any sub-category thereof, were considered eligible for inclusion. For a resource on research-validated scales for the measurement of suicidality we relied on the list compiled by Ghasemi, Shaghaghi, and Allahverdipour.^33^ We sought to identify recently described, effective treatments for the prevention of suicidal behavior that are outside the state-of-the-art (current standards). To this end, we defined recent interventions as being patient-centered, delivered in the ED, described within the past 10 years, and not already part of recognized state-of-the-art practice.

### Features of Eligible Studies

We assessed studies for characteristic features once they were included in the analysis, and we evaluated the comparative strengths and weaknesses of study design, sample size, etc. Studies considered identified a specific, recent intervention for acute suicidality in context of the ED in the previous 10 years, since earlier interventions were considered more likely to be consistent with state-of-the-art practice.

#### Search strategy

We used a three-step search strategy in consultation with a library scientist. In the first phase, we conducted a preliminary search of PubMed to ensure relevant results were retrieved from our search terms. In the second phase, the search terms were applied to PubMed, SCOPUS, and CINAHL. See Appendix A for the search terms used. In the third phase, we scanned the results from the search conducted in phase two for references included in study bibliographies that could have provided additional articles. The database search strategy is summarized in Appendix A. We conducted additional searches of Google to identify gray literature or publications not discovered via the above-described search process.

#### Study selection

Once search terms and keywords were narrowed down, we removed duplicates from the list of articles. Four independent reviewers screened the titles and abstracts for relevance of all remaining studies. Articles determined to be relevant at this stage were retrieved in full-text form and screened for relevance by two independent reviewers. A pre-selected arbiter settled inconsistencies between reviewers. We recorded and documented reasons for exclusion for any article. A visualization of this process is included in a PRISMA flow diagram^34^ in Figure 1, which also provides a a summary of results in standard PRISMA format.

**Figure.**
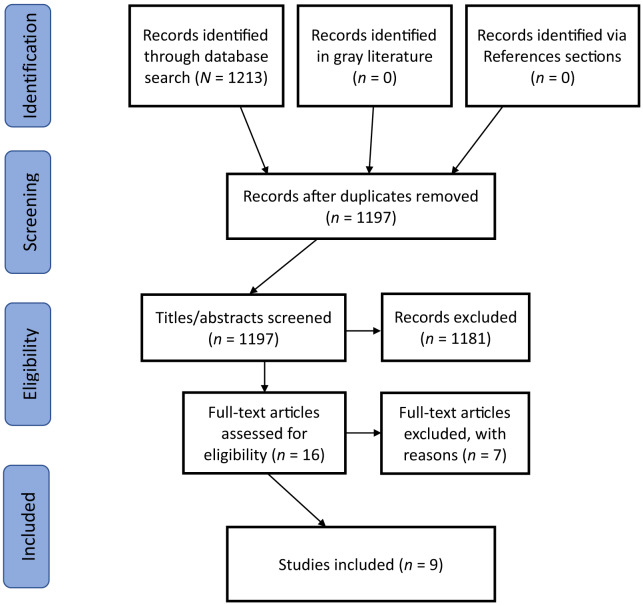
PRISMA flow diagram of study selection.

#### Data extraction

Data fields collected from included studies are summarized in the data extraction tool given in Appendix B. The primary author A.P.H. extracted data using the tool, and the data was checked for accuracy and completeness by an independent reviewer.

## RESULTS

After duplicates were removed, we analyzed 1,197 studies. There are a few reasons for this large number of results. In keeping with best-practice guidelines, and to avoid the improper exclusion of any relevant articles, we used broad search terms to return the maximum number of potentially relevant articles. Additionally, no MeSH terms specifically aimed at excluding screening and risk assessment were used. The title/abstract review phase was, therefore, a critical stage for the isolation of relevant articles, and we removed 1,181 from further analysis.

In the next phase of eligibility screening, two independent reviewers retrieved and evaluated the full texts of 16 articles, of which seven were excluded. Disagreements were settled by an emergency physician with relevant expertise in acute care interventions for suicidality R.A.D. Of the excluded articles, two studies involved interventions that were tailored to the unique cultural practices of specific indigenous groups and were therefore deemed not generalizable to all suicidal patients presenting to EDs.^35^ ^,^ ^36^ Two additional articles were excluded as they used a safety planning intervention operationally defined as part of state-of-the-art practice. One article was excluded because the study intervention did not occur in the ED setting.^37^ Finally, we excluded two secondary analyses of articles that had already been included.^38^ ^,^ ^39^ Of the nine articles included in the analysis, we extracted data using the tool given in Appendix A. An overview of relevant data from each study is presented in the Table.

**Table.** Summary of individual studies.

Authors	Year	Country	Objective(s)	Intervention	Methods	N	Length of time	Discipline	Materials	Follow-up	Findings	Conclusion
Dana Alonzo	2014	USA	To determine the feasibility and acceptability of a novel, manualized problem-solving and comprehensive contact intervention (PS–CCI) aimed at improving treatment engagement of suicidal individuals.	Manualized problem-solving and comprehensive contact intervention	Longitudinal design	44	3 mos	“Clinician” not otherwise defined	Structured worksheet manualized	3 mos	Did not have the sample for statistical significance, but they appeared to find that this intervention was feasible as measured by acceptability, engagement, fidelity, subject retention, and lack of AEs.	PS-CCI is feasible as an intervention for suicide in the ED
Kawashini et al	2014	Japan	To determine if assertive case management can reduce repeated suicide attempts in people with mental health problems who had attempted suicide and were admitted to EDs	Assertive case management	Two-armed RCT	914	18 mos to 5 years	Psychiatrist, nurses, clinical psychologists, etc	Case management manual, phone	18 mos-5 yrs	Lower recurrent suicidal behavior at 1, 3, and 6 mos; but at 18 mos there was no significant difference in rates of attempted or completed suicides	Intervention somewhat effective short term and among women, but overall assertive case management did not outperform control condition
Grupp-Phelan et al	2019	USA	To examine whether motivational interviewing (MI) increases linkage of adolescents to outpatient mental health services for depression and suicide risk	MI plus linking to follow up services	Two-armed RCT	159	2 mos total	Licensed social worker	None	6 mos	MI was not better than enhanced usual care	Brief MI was not useful in this context
Bryan et al	2017	USA	To evaluate the effectiveness of crisis response planning (CRP) for the prevention of suicide attempts	CRP	Three-armed RCT	97	1 hr	Clinician not defined	Index card	6 mos	Standard CRP and reasons for living CRP were both more effective than a contract for safety in reducing suicide attempts and inpatient hospitalization. No difference between two versions of CRP	CRP is highly effective for use in the acute care setting for acutely suicidal patients
Domany and McCullums Smith	2020	USA	To assess the safety, feasibility, tolerability, and efficacy of a single low-dose intravenous (IV) ketamine in reducing suicidal ideation	Ketamine IV	Placebo-controlled double blind RCT	18	5 mins	Board-certified ED physician	IV bag, drug, 10ml syringe, vital sign monitoring equipment	Two weeks	Significant differences in suicidality scores 90–180 mins after IV ketamine infusion. Safety and tolerability were excellent.	IV ketamine appears to be feasible and effective in the short term
Burger et al	2016	USA	To compare IV ketamine to placebo among acutely suicidal patients in a military setting	Ketamine IV	Double-blind RCT	10	5 mins	Trained nurse	IV bag, vital sign monitoring equipment	Two weeks	Groups varied significantly in the short-term during hospitalization; however, discrepancies in scores on the rating scales diminished at the two-week follow-up point.	Ketamine shows promise as an acute care treatment modality, but further research is needed to validate the effects of this very small trial.
King et al	2015	USA	This randomized trial examined the effectiveness of Teen Options for Change (TOC), an intervention for adolescents seeking medical emergency services who screen positive for suicide	Teen Options for Change (TOC)	RCT with enhanced TAU as control condition	49	45 mins of MI, 5 days of follow up	Trained mental health professional with minimum of 40 hrs of specialized training	Handwritten follow up note	2 mos	Large effect for the lowing of depression scores, moderate effect for hopelessness, and insignificant effects for suicidal ideation and alcohol use (though the study wasn’t quite large enough to statistically verify the latter findings	This intervention appears to be promising but will need to be tested in a larger sample
Kashani et al	2014	Iran	This study was conducted to examine the effects of a single intravenous bolus of ketamine on patients with suicidal ideation in the ED.	Ketamine IV bolus	Uncontrolled pre-test/post-test	49	5 mins	Nurse or attending doctor	IV bag, vital sign- monitoring equipment	10 days	The ketamine infusion was associated with a bringing down the MADRS and SSI scores by a statistically significant margin, but the authors note that, for their purposes, the reduction in scores was not clinically meaningful (e.g., below the threshold set for the purposes of the study of a MADRS score of ≤4). The largest reduction in self-reported suicidality occurred within 40 mins of ketamine administration.	The authors note that ketamine did not show itself as a promising tool to mitigate acute suicidality in the ED context, as the reductions in suicidality scores were not clinically meaningful. However, they do note that more research is needed and that this result is far from definitive (given the sample size and lack of control group).
Domany and McCullum Smith	2022	USA	Evaluation of single, fixed-dosed intranasal ketamine for acute suicidal ideation in the ED	Ketamine intranasal	Double-blind RCT	30	40 mins to 1 hr	Psychiatrist	Intranasal atomizer	4 weeks	Participants who were administered ketamine had not only a much higher rate of remission (roughly 80% of the sample), but also typically shorter lengths of hospitalization. Ketamine was generally safe; minor side effects were transient and resolved within 1–2 hours post-administration. However, the linear mixed statistical model did not show significance on random group by time interaction.	While the sample is comparatively small and the overall results are far from conclusive, this study importantly echoes previous findings demonstrating that a low dose of ketamine does appear to transiently reduce suicidal ideation in those at high acute risk for suicide. In addition, it provides promising evidence that a single, fixed dose of ketamine may have the potential to reduce hospital lengths of stay, and that intranasal is a feasible route of administration for the acute care setting.

*AEs*, adverse events; *ED*, emergency department; *IV*, intravenous; *MADRS*, Montgomery Asberg depression rating scale; *RCT*, randomized, controlled trial; *SSI*, Beck Scale for Suicidal Ideation; *TAU*, treatment as usual.

Four included articles assessed the use of a single dose of the pharmacologic intervention ketamine, a N-methyl-D-aspartate antagonist commonly used as a sedative, analgesic, and anesthetic.^40^ ^–^ ^43^ Three studies assessed the use of an intravenous (IV) infusion,^40^ ^,^ ^42^ ^,^ ^43^ and one assessed the efficacy and tolerability of an intranasal administration.^41^ Two of the selected articles assessed the efficacy of interventions centered on motivational interviewing (MI) embedded in an interventional framework with provisions for follow-up care or referral assistance.^44^ ^,^ ^45^ Both Teen Options for Change (TOC)^45^ and Suicidal Teens Accessing Treatment After an Emergency Department Visit (STAT-ED)^44^ targeted adolescent samples.

The three remaining included articles studied various interventions centered on acute psychotherapy and/or behavioral management in the post-acute period. By far the largest sample among included articles was a study of assertive case management for those presenting to the ED after a suicide attempt.^46^ While the lengthy (18+ months) intervention under study in this article largely took place following discharge from the ED, the intervention procedures began while patients were in the ED and were delivered by psychiatrists or other medical personnel.^46^ Another study that met our criteria investigated the efficacy of the novel, manualized Problem Solving and Comprehensive Contact Intervention (PS-CCI), which uses a collaboratively completed worksheet aimed at enhancing self-efficacy and cognitive flexibility in suicidal ED patients paired with follow-up care.^47^ Finally, a study of the Crisis Response Plan (CRP) intervention conducted by Bryan and colleagues in a military ED met inclusion criteria.^22^ The CRP pairs a brief historical interview with a collaborative identification and documentation of coping strategies and resources available to patients.^22^

### Study Features and Characteristics

Several measures of study features and characteristics were gathered in the process of data extraction. We used PRISMA guidelines to help define elements of quality^31^ including sample size, study design, follow-up timeframe, and measures used.

#### Sample

The sample sizes of the majority of studies meeting inclusion criteria were small. Excluding the outlier of Kawanishi et al^46^ with their robust sample of 914, the average sample size for included studies was 57, with the smallest sample (10) collected by Burger and colleagues.^40^

#### Design

Seven of nine studies conducted a randomized controlled trial (RCT), one was a quasi-experimental two-arm prospective longitudinal study,^47^ and one was a non-experimental pilot study designed to evaluate the feasibility and efficacy of a single low dose of ketamine delivered via IV bolus.^43^ Double-blinding and random assignment were consistently practiced among the RCTs assessing the efficacy of ketamine, and participants in the control conditions received an inactive placebo injection/atomization of normal saline.^40^ ^–^ ^42^ Kawanishi and colleagues,^46^ King et al,^45^ and Grupp-Phelan et al^44^ used single blinding and a comparator condition of enhanced usual care (EUC). In their three-arm RCT, Bryan and colleagues^22^ compared two versions of CRP (standard and enhanced) to the control condition of a contract for safety, and participants were blinded to group assignment. Although the contract for safety was previously a standard intervention, it has many noteworthy shortcomings^22^ making it a less-than-ideal comparison condition to CRP.^24^

#### Follow-up measures and timeframe

Seven of the nine studies included in this review used standard, well-subscribed, psychometrically validated measures of suicidality to assess outcome variables of interest, as well as evaluations of repeated hospitalizations and healthcare utilization. The most common scales used were the Columbia Suicide Severity Rating Scale, the Beck Scale for Suicidal Ideation, and the Montgomery-Asberg Depression Rating Scale. However, two studies evaluated only post-discharge suicide attempts, suicide deaths, and psychiatric hospitalization recidivism without making use of psychometric measures.^46^ ^,^ ^47^ The follow-up timeframes varied significantly depending on the intervention under study and added to the heterogeneity of the sample.

### Fit of Intervention to Emergency Department Environment

Since we intended to analyze recently described tools available to emergency physicians for use in the acute care setting, attention was paid to the usability of each intervention in the ED setting. We defined usability as ease of use and fit to the ED environment, and these were evaluated along the dimensions of total time required to administer, the required training or credentials of the administering practitioner, and the tools and materials required to deliver the intervention.

#### Time to administer

By far the briefest interventional modality among the articles reviewed was a single dose of ketamine delivered IV, which, at the modest doses used, averaged 5–10 minutes.^40^ ^,^ ^42^ ^,^ ^43^ When administered intranasally, the interval required to complete the intervention, although brief (40–60 minutes), was somewhat longer.^41^ Not included in the intervention duration was the monitoring time required for ketamine administration, which depending on local protocol can exceed several hours. Equivalently brief is CRP, which requires 30–60 minutes to administer, making it well-suited to the demands of the fast-paced ED environment.^22^ The studies assessing MI-based interventions for adolescents had brief ED-delivered components, requiring approximately 45 minutes to deliver.^44^ ^,^ ^45^ The time required to administer the ED-based problem-solving component of the Problem-Solving and Comprehensive Contact Intervention (PS-CCI) intervention was not specified.^47^ Finally, Kawanishi and colleagues did not specify the time course of the ED-based portion of assertive case management, but they did note the intervention involved regular follow-up appointments outside the ED over the course of 18 months.^46^

#### Training required to administer

Due in part to variability in hospital practices in different regions and countries, the credentials of the healthcare professionals administering ketamine varied slightly across the four studies that investigated its use.^40^ ^–^ ^43^ Intravenous administration was conducted by either a nurse or a physician,^40^ ^,^ ^42^ ^,^ ^43^ whereas the study using intranasal ketamine required significant input from a pharmacist.^41^ Both the PS-CCI^47^ and CRP^22^ stated only that the intervention was delivered by a “clinician,” not otherwise specified. The STAT-ED described by Grupp-Phelan et al^44^ and TOC studied by King et al^45^ were administered by a social worker and trained mental health professional, respectively, with the latter specifying that interventionalists were required to have a minimum of 40 hours of specialized training. Finally, the assertive case management intervention described by Kawanishi and colleagues^46^ was conducted by case managers at various levels of training, including nurses, emergency physicians, psychiatrists, and clinical psychologists.

#### Tools and materials

For most psychosocial interventions under study in the present review, few specialized materials were required for administration. Specifically, the STAT-ED intervention,^44^ CRP,^22^ and TOC^45^ require basic office equipment such as copy paper and notecards. The PS-CCI intervention requires the availability of a structured worksheet,^47^ and the assertive case management intervention requires a standardized manual,^46^ making their resource demands minimal. As with all novel pharmacologic interventions, the studies assessing a single dose of ketamine required the availability of equipment to monitor vital signs.^40^ ^–^ ^43^ Those assessing IV ketamine required IV bags, pumps, lines, and hanging apparatuses, which are usually available in ED environments,^40^ ^,^ ^42^ ^,^ ^43^ while the study of intranasal ketamine required a specialized atomizer prepared by a pharmacy team.^41^

### Efficacy Findings

The interpretation of findings for articles described in the present review should be moderated by limitations regarding sample size, methodological discrepancies, and evidentiary quality. Two promising interventions we identified are the various administration routes of a single, low dose of ketamine,^40^ ^–^ ^43^ and a single meeting to develop a CRP.^22^ For a single dose of ketamine, three articles reported positive findings on the short-term reduction of self-reported suicidality and depression,^40^ ^–^ ^42^ and one reported inconclusive results.^43^ Bryan and colleagues^22^ found that participants randomized to either CRP condition (standard or enhanced) showed significant reductions in acute suicidal ideation, fewer suicide attempts, and lower rates of inpatient hospitalization post-discharge than those in the comparator group.

Two other interventions that were evaluated, PS-CCI and MI, show promise, but there is insufficient evidence to support their efficacy. The PS-CCI trial^47^ was not statistically powered to determine efficacy, but the authors note that the intervention is feasible to administer in the ED setting given its high tolerability. The TOC study^45^ and the STAT-ED study^44^ trialed similar MI-based treatments in comparable adolescent samples but returned conflicting results. The study of assertive case management by Kawanishi and colleagues^46^ had a large sample size but demonstrated no significant difference between groups over the course of the study.

## DISCUSSION

The preliminary results from the four ketamine studies included in this review echo findings of the use of ketamine for suicidal ideation in outpatient settings.^48^ There are a number of advantages to this interventional modality.^43^ First, ketamine, when administered IV over 5–10 minutes, is by far the briefest intervention not considering the post-infusion monitoring time. Intravenous ketamine is well suited to the fast-paced environment of the ED. The intranasal administration route is almost as brief. Intramuscular (IM) ketamine is another option but relatively unstudied; however, it may be familiar to emergency clinicians. If confirmed in fully powered RCTs, such a rapid-acting intervention may give emergency physicians additional options for the placement or even discharge of patients who present with acutely elevated suicide risk and could serve as a bridge to definitive mental health care that circumvents the need for a lengthy detention in the ED. Furthermore, a dose of generic ketamine is relatively inexpensive^49^ and regularly stocked in most EDs. The intranasal form of ketamine, esketamine, in contrast, is more expensive and less widely stocked. Additional research on the efficacy of ketamine for acutely suicidal ED patients is warranted.

This review found evidence that CRP shows promise as an intervention well suited to combat acute suicidality in the ED environment. While there is limited evidence in support of the efficacy of CRP in the ED, this intervention has several features that make it well suited to the specific demands on emergency medical personnel. First, similar to a single dose of ketamine, CRP is an interventional modality that is brief in administration and appears to rapidly diminish acute suicidality and improve patient mood.^38^ Additionally, CRP is maximally portable to a variety of environments, requires comparatively little specialized training, tools or materials to administer, and, as a psychosocial intervention, is not contraindicated for use with any concomitant medications. Despite its advantages, the literature to date on the use of CRP in the ED context is limited to one study.^22^ While the evidence for interventions such as the PS-CCI^47^ and TOC^45^ is mixed, ED-delivered interventions targeting constructs of cognitive flexibility and adaptive problem-solving appear to be a recipe with some promise (similar to CRP).

### Future Directions

This study identified two promising interventions suited to the ED environment: CRP and ketamine. The evidentiary basis for these interventions, particularly in broad-based populations of emergently suicidal ED patients, is not fully developed. Further study is required to ascertain the extent to which these interventions serve as effective treatments across presenting psychiatric symptoms, especially given the high incidence of SRTB among patients with serious mental illness or acute intoxication with a substance, which may complicate effective treatment.^50^ Given the crisis of boarding in EDs, additional funding and study in general should be a national priority. Future studies should also investigate ketamine delivered via alternative routes of administration such as orally and IM. While CRP has demonstrated preliminary efficacy,^24^ future research should compare CRP to validated current standard practice interventions to properly evaluate its effectiveness against treatment as usual or EUC. Future studies should also validate use of the intervention outside the military context with participants of various backgrounds, ability levels, and ages. Given that brief MI- and CBT-based interventions show promise, future studies may consider continuing to hone interventions that approximately adhere to this model.

## LIMITATIONS

Although the present study has many notable strengths, some shortcomings should be delineated. First, we focused on interventions with evidence supporting the ability to be performed in the challenging ED environment. It is possible that recent interventions under study in other clinical environments may hold promise for adaptation to the ED setting. Second, as is the case with any review, it is possible that certain interventions extant in the literature were erroneously excluded from our analysis given the limitations of our MeSH search terms. Finally, to limit our analysis to only the most recent interventions with an evidentiary basis in the current literature, we assessed only articles published in the previous 10 years. It is possible that there are promising, ED-based interventions described more than 10 years ago that have received no further study in the intervening time or have been studied exclusively outside the ED context since their initial description.

## CONCLUSION

The recently described interventions identified for emergency physicians to treat acute suicidality are limited to one drug (ketamine) and four unique psychological/behavioral interventions. Two of the five interventional modalities have preliminary evidence and may hold promise in mitigating acute suicide risk in the ED: a single, low dose of ketamine and crisis response planning. However, there is insufficient evidence to support their widespread adoption. Future research should extend the preliminary findings summarized in this review.

## Supplementary Information






## References

[1] Stone DM Simon TR Fowler KA et al . Vital signs: trends in state suicide rates - United States, 1999–2016 and circumstances contributing to suicide - 27 states, 2015. Morb Mortal Wkly Rep. 2018;67(22):617–24. 10.15585/mmwr.mm6722a1 PMC5991813 29879094

[2] Ahmedani BK Simon GE Stewart C et al . Health care contacts in the year before suicide death. J Gen Intern Med. 2014;29(6):870–7. 10.1007/s11606-014-2767-3 PMC4026491 24567199

[3] Gairin I House A Owens D . Attendance at the accident and emergency department in the year before suicide: retrospective study. Br J Psychiatry. 2003;183:28–33. 12835240 10.1192/bjp.183.1.28

[4] Crandall C Fullerton-Gleason L Aguero R et al . Subsequent suicide mortality among emergency department patients seen for suicidal behavior. Acad Emerg Med. 2006;13(4):435–42. 16531601 10.1197/j.aem.2005.11.072

[5] Weiss AJ Barrett ML Heslin KC et al . (2006). Trends in emergency department visits involving mental and substance use disorders, 2006–2013. In Healthcare Cost and Utilization Project statistical briefs. Rockville, MD: Agency for Healthcare Research and Quality (US). Available at: https://www.ncbi.nlm.nih.gov/books/NBK52651/. Accessed August 6, 2024. 28121114

[6] Hakenewerth AM . Emergency department visits by patients with mental health disorders--North Carolina, 2008–2010. Morb Mortal Wkly Rep. 2013;62(23):469–72. PMC4604846 23760188

[7] Owens C Hansford L Sharkey S et al . Needs and fears of young people presenting at accident and emergency department following an act of self-harm: secondary analysis of qualitative data. Br J Psychiatry. 2016;208(3):286–91. 26450583 10.1192/bjp.bp.113.141242 PMC4807637

[8] Treatment Advocacy Center . Going, going, gone: trends and consequences of eliminating state psychiatric beds. 2016. Available at: https://www.treatmentadvocacycenter.org/reports_publications/going-going-gone-trends-and-consequences-of-eliminating-state-psychiatric-beds/. Accessed September 4, 2023.

[9] Gold J . A dearth of hospital beds for patients in psychiatric crisis. 2016. Available at: https://kffhealthnews.org/news/a-dearth-of-hospital-beds-for-patients-in-psychiatric-crisis/. Accessed August 6, 2024.

[10] Zun L . Care of psychiatric patients: the challenge to emergency physicians. West J Emerg Med. 2016;17(2):173–6. 26973743 10.5811/westjem.2016.1.29648 PMC4786237

[11] Fitzpatrick SJ River J . Beyond the medical model: future directions for suicide intervention services. Int J Health Serv. 2018;48(1):189–203. 28649928 10.1177/0020731417716086

[12] Nicks BA Manthey DM . The impact of psychiatric patient boarding in emergency departments. Emerg Med Int. 2012;2012:360308. 22888437 10.1155/2012/360308 PMC3408670

[13] Rihmer ZP Maurizio P .Mood disorders: suicidal behavior. In: Sadock BJ (Ed.), Kaplan and Sadock’s Comprehensive Textbook of Psychiatry (3825), 10th ed. Philadelphia, PA: Wolters Kluwer Health, 2017:3825–51.

[14] Wasserman D . Suicide: overview and epidemiology. In: Sadock BJ (Ed.), Kaplan and Sadock’s Comprehensive Textbook of Psychiatry (5954). 10th ed. Philadelphia, PA: Wolters Kluwer Health, 2017:5954–72.

[15] Capoccia LM . Caring for adult patients with suicide risk: a consensus-based guide for emergency departments. 2015. Available at: https://sprc.org/wp-content/uploads/2022/11/EDGuide_full.pdf. Accessed August 6, 2024.

[16] Betz J.M . Suicide. In: Rosen’s Emergency Medicine: Concepts and Clinical Practice (page range). 9th ed. Philidelphia, PA: Elsevier, Inc, 2018:1366–73.

[17] Sudak H . Suicide treatment. In: Sadock B.J. (Ed.), Kaplan and Sadock’s Comprehensive Textbook of Psychiatry (5973). 10th ed. Philadelphia, PA: Wolters Kluwer, 2017:5973–85.

[18] Reeves RR Ladner ME . Antidepressant-induced suicidality: an update. CNS Neurosci Ther. 2010;16(4):227–34. 20553304 10.1111/j.1755-5949.2010.00160.x PMC6493906

[19] Rudd MD Bryan CJ Wertenberger EG et al . Brief cognitive-behavioral therapy effects on post-treatment suicide attempts in a military sample: results of a randomized clinical trial with 2-year follow-up. Am J Psychiatry. 2015;172(5):441–9. 25677353 10.1176/appi.ajp.2014.14070843

[20] Davidson KM Brown TM James V et al . Manual-assisted cognitive therapy for self-harm in personality disorder and substance misuse: a feasibility trial. Psychiatr Bull (2014). 2014;38(3):108–11. 25237519 10.1192/pb.bp.113.043109 PMC4115373

[21] Chang BP Tezanos K Gratch I et al . Depressed and suicidal patients in the emergency department: an evidence-based approach. Emerg Med Pract. 2019;21(5):1–24. 31033267

[22] Bryan CJ Mintz J Clemans TA et al . Effect of crisis response planning vs. contracts for safety on suicide risk in U.S. Army soldiers: a randomized clinical trial. J Affect Disord. 2017;212:64–72. 28142085 10.1016/j.jad.2017.01.028

[23] Shin HD Cassidy C Weeks LE et al . Interventions to change clinicians’ behavior in relation to suicide prevention care in the emergency department: a scoping review protocol. JBI Evid Synth. 2021;19(8):2014–23. 33795582 10.11124/JBIES-20-00307

[24] Rudd MD Mandrusiak M Joiner TE . The case against no-suicide contracts: the commitment to treatment statement as a practice alternative. J Clin Psychol. 2006;62(2):243–51. 16342293 10.1002/jclp.20227

[25] Inagaki M Kawashima Y Kawanishi C et al . Interventions to prevent repeat suicidal behavior in patients admitted to an emergency department for a suicide attempt: a meta-analysis. J Affect Disord. 2015;175:66–78. 25594513 10.1016/j.jad.2014.12.048

[26] Mann JJ Michel CA Auerbach RP . Improving suicide prevention through evidence-based strategies: a systematic review. Am J Psychiatry. 2021;178(7):611–24. 33596680 10.1176/appi.ajp.2020.20060864 PMC9092896

[27] Johnston AN Spencer M Wallis M et al . Review article: Interventions for people presenting to emergency departments with a mental health problem: a systematic scoping review. Emerg Med Australas. 2019;31(5):715–29. 31257713 10.1111/1742-6723.13335

[28] Lengvenyte A Olié E Strumila R et al . Immediate and short-term efficacy of suicide-targeted interventions in suicidal individuals: a systematic review. World J Biol Psychiatry. 2021;22(9):670–85. 33783294 10.1080/15622975.2021.1907712

[29] Marx JA Hockberger RS Walls RM et al . In: Rosen’s Emergency Medicine: Concepts and Clinical Practice. 9th ed. Philadelphia, PA: Mosby/Elsevier, 2018.

[30] Sadock BJS Alcott V Ruiz P . Kaplan and Sadock’s Comprehensive Textbook of Psychiatry. 10th ed. Philadephia, PA: Wolters Kluwer, 2017.

[31] Tricco AC Lillie E Zarin W et al . PRISMA extension for scoping reviews (PRISMA-ScR): checklist and explanation. Ann Intern Med. 2018;169(7):467–73. 30178033 10.7326/M18-0850

[32] Silverman MM Berman AL Sanddal ND et al . Rebuilding the Tower of Babel: a revised nomenclature for the study of suicide and suicidal behaviors. Part 2: suicide-related ideations, communications, and behaviors. Suicide Life Threat Behav. 2007;37(3):264–77. 17579539 10.1521/suli.2007.37.3.264

[33] Ghasemi P Shaghaghi A Allahverdipour H . Measurement scales of suicidal ideation and attitudes: a systematic review article. Health Promot Perspect. 2015;5(3):156–68. 26634193 10.15171/hpp.2015.019 PMC4667258

[34] Moher D Liberati A Tetzlaff J et al . Preferred reporting items for systematic reviews and meta-analyses: the PRISMA statement. PLoS Med. 2009;6(7):e1000097. 19621072 10.1371/journal.pmed.1000097 PMC2707599

[35] Cwik MF Tingey L Lee A et al . Development and piloting of a brief intervention for suicidal American Indian adolescents. Am Indian Alsk Native Ment Health Res. 2016;23(1):105–24. 28562844 10.5820/aian.2301.2016.105

[36] Hatcher S Coupe N Wikiriwhi K et al . Te Ira Tangata: a Zelen randomised controlled trial of a culturally informed treatment compared to treatment as usual in Māori who present to hospital after self-harm. Soc Psychiatry Psychiatr Epidemiol. 2016;51(6):885–94. 26956679 10.1007/s00127-016-1194-7

[37] Miller IW Camargo CA Jr. Arias SA et al . Suicide prevention in an emergency department population: the ED-SAFE study. JAMA Psychiatry. 2017;74(6):563–70. 28456130 10.1001/jamapsychiatry.2017.0678 PMC5539839

[38] Bryan CJ Mintz J Clemans TA et al . Effect of crisis response planning on patient mood and clinician decision making: a clinical trial with suicidal U.S. soldiers. Psychiatr Serv. 2018;69(1):108–11. 28967323 10.1176/appi.ps.201700157

[39] Furuno T Nakagawa M Hino K et al . Effectiveness of assertive case management on repeat self-harm in patients admitted for suicide attempt: findings from ACTION-J study. J Affect Disord. 2018;225:460–5. 28863298 10.1016/j.jad.2017.08.071

[40] Burger J Capobianco M Lovern R et al . A double-blinded, randomized, placebo-controlled sub-dissociative dose ketamine pilot study in the treatment of acute depression and suicidality in a military emergency department setting. Mil Med. 2016;181(10):1195–9. 27753551 10.7205/MILMED-D-15-00431

[41] Domany Y McCullumsmith CB . Single, fixed-dose intranasal ketamine for alleviation of acute suicidal ideation. An emergency department, trans-diagnostic approach: a randomized, double-blind, placebo-controlled, proof-of-concept trial. Arch Suicide Res. 2022;26(3):1250–65. 33583341 10.1080/13811118.2021.1878078

[42] Domany Y Shelton RC McCullum Smith CB . Ketamine for acute suicidal ideation. An emergency department intervention: A randomized, double-blind, placebo-controlled, proof-of-concept trial. Depress Anxiety. 2020;37(3):224–33. 31733088 10.1002/da.22975

[43] Kashani P Yousefian S Amini A et al . The effect of intravenous ketamine in suicidal ideation of emergency department patients. Emerg (Tehran). 2014;2(1):36–9. 26495340 PMC4614623

[44] Grupp-Phelan J Stevens J Boyd S et al . Effect of a motivational interviewing-based intervention on initiation of mental health treatment and mental health after an emergency department visit among suicidal adolescents: a randomized clinical trial. JAMA Netw Open. 2019;2(12):e1917941. 31860104 10.1001/jamanetworkopen.2019.17941 PMC6991223

[45] King CA Gipson PY Horwitz AG et al . Teen options for change:an intervention for young emergency patients who screen positive for suicide risk. Psychiatr Serv. 2015;66(1):97–100. 25321886 10.1176/appi.ps.201300347 PMC4346207

[46] Kawanishi C Aruga T Ishizuka N et al . Assertive case management versus enhanced usual care for people with mental health problems who had attempted suicide and were admitted to hospital emergency departments in Japan (ACTION-J): a multicentre, randomised controlled trial. Lancet Psychiatry. 2014;1(3):193–201. 26360731 10.1016/S2215-0366(14)70259-7

[47] Alonzo D . Suicidal individuals and mental health treatment: a novel approach to engagement. Community Ment Health J. 2016;52(5):527–33. 26748654 10.1007/s10597-015-9980-3

[48] Xiong J Lipsitz O Chen-Li D et al . The acute antisuicidal effects of single-dose intravenous ketamine and intranasal esketamine in individuals with major depression and bipolar disorders: a systematic review and meta-analysis. J Psychiatr Res. 2021;134:57–68. 33360864 10.1016/j.jpsychires.2020.12.038

[49] Brendle M Robison R Malone DC . Cost-effectiveness of esketamine nasal spray compared to intravenous ketamine for patients with treatment-resistant depression in the US utilizing clinical trial efficacy and real-world effectiveness estimates. J Affect Disord. 2022;319:388–96. 36162672 10.1016/j.jad.2022.09.083

[50] Rabasco A Arias S Benz MB et al . Longitudinal risk of suicide outcomes in people with severe mental illness following an emergency department visit and the effects of suicide prevention treatment. J Affect Disord. 2024;347:477–85. 38065475 10.1016/j.jad.2023.12.019 PMC10872614

