# Supplementary material for: Recent Interventions for Acute Suicidality Delivered in the Emergency Department: A Scoping Review
Source: West J Emerg Med. 2024 Oct 9;25(6):858–68. doi: 10.5811/westjem.18640 (PMC11610724; doi:10.5811/westjem.18640)
Supplement: Supplementary file 1 [file wjem-25-858-s001.docx]

Appendix A: Sample Search Strategy

**Search Strategy for PubMed**

| **Search#** | **Query** | **Results** |
| --- | --- | --- |
| 6 | (#4) AND (("2013/01/01"[Date - Publication] : "3000"[Date - Publication])) | **753** |
| 5 | (#4) AND (("2008/01/01"[Date - Publication] : "3000"[Date - Publication])) | **936** |
| 4 | #1 AND #2 AND #3 | 1,145 |
| 3 | ((Crisis Intervention[Mesh]) OR (Risk Assessment[Mesh])) OR (Crisis Intervention[tiab] OR Crisis Interventions[tiab] OR Brief Advice[tiab] OR Screening and Brief Intervention[tiab] OR Brief Treatment[tiab] OR Brief Treatments[tiab] OR Brief Intervention[tiab] OR Brief Interventions[tiab] OR Critical Incident Stress Debriefing[tiab] OR interven*[tiab] OR screen*[tiab] OR Risk assessment[tiab] OR Risk assessments[tiab] OR "Ask Suicide Screening Questions"[tiab] OR ASQ[tiab] OR suicide scan[tiab] OR suicide-scale[tiab] OR C-SSRS[tiab] OR depression-scale[tiab] OR questionnaire[tiab] OR PHQ-9[tiab] OR prevention-resources[tiab] OR PSS-3[tiab] OR Risk assessment tools[tiab] OR SAFE-T[tiab] OR SBQ-R[tiab] OR screening-tool[tiab] OR screening-tools[tiab] OR SSI-W[tiab] OR suicide-questionnaire[tiab] OR tools[tiab] OR instruments[tiab]) | 3,006,586 |
| 2 | ((Suicide[Mesh]) OR (Self-Injurious Behavior[Mesh])) OR (Suicidal ideation[tiab] OR Suicidal ideations[tiab] OR Self Injurious Behavior[tiab] OR Self-Injurious Behaviors[tiab] OR Intentional Self Injury[tiab] OR Intentional Self Injuries[tiab] OR Intentional Self Harm[tiab] OR Nonsuicidal Self Injury[tiab] OR Nonsuicidal Self Injuries[tiab] OR Deliberate Self-Harm[tiab] OR Deliberate Self Harm[tiab] OR Self-Injury[tiab] OR Self Injury[tiab] OR Self Harm[tiab] OR Self-Destructive Behavior[tiab] OR Self Destructive Behavior[tiab] OR Self-Destructive Behaviors[tiab]) | 86,248 |
| 1 | (Emergency Service, Hospital[Mesh]) OR (Hospital Emergency Services[tiab] OR Emergency Hospital Service[tiab] OR Emergency Hospital Services[tiab] OR Hospital Service Emergency[tiab] OR Hospital Service Emergencies[tiab] OR Emergency Units[tiab] OR Emergency Unit[tiab] OR Accident and Emergency Department[tiab] OR Emergency Ward[tiab] OR Emergency Wards[tiab] OR Emergency Departments[tiab] OR Emergency Department[tiab] OR Emergency Room[tiab] OR Emergency Rooms[tiab] OR Emergency Outpatient Unit[tiab] OR Emergency Outpatient Units[tiab]) | 171,110 |
