# Supplementary material for: Recent Interventions for Acute Suicidality Delivered in the Emergency Department: A Scoping Review
Source: West J Emerg Med. 2024 Oct 9;25(6):858–68. doi: 10.5811/westjem.18640 (PMC11610724; doi:10.5811/westjem.18640)
Supplement: Supplementary file 2 [file wjem-25-858-s002.docx]

Appendix B: Data Extraction Tool.

| Reviewer initial: | Date: |
| --- | --- |
| Title | |
|  | |
| Year of publication | |
|  | |
| Authors | |
|  | |
| Country of origin | |
|  | |
| Study objective(s) | |
|  | |
| Methods | |
|  | |
| Sample size | |
|  | |
| Sample characteristics/demographics | |
|  | |
| Setting | |
|  | |
| Age of sample (adult or pediatric) | |
|  | |
| Intervention description (drug, psycho/behavioral) | |
|  | |
| Intervention described as novel, new, original, groundbreaking, etc. (Y/N) | |
|  | |
| Length of time for administration of intervention | |
|  | |
| Professional administering intervention/training required to administer | |
|  | |
| Tools and material required to administer intervention | |
|  | |
| Other notes on intervention | |
|  | |
| Measures | |
|  | |
| Follow up period/timeframe | |
|  | |
| Findings | |
|  | |
| Conclusion | |
|  | |
